# OptimalMe Intervention for Healthy Preconception, Pregnancy, and Postpartum Lifestyles: Protocol for a Randomized Controlled Implementation Effectiveness Feasibility Trial

**DOI:** 10.2196/33625

**Published:** 2022-06-09

**Authors:** Cheryce L Harrison, Bonnie R Brammall, Rhonda Garad, Helena Teede

**Affiliations:** 1 Monash Centre for Health Research and Implementation School of Public Health and Preventive Medicine Clayton Australia; 2 Endocrine and Diabetes Unit Monash Health Clayton Australia

**Keywords:** preconception, pregnancy, postpartum, weight, obesity prevention, womens health, intervention, implementation

## Abstract

**Background:**

Reproductive-aged women are a high-risk population group for accelerated weight gain and obesity development, with pregnancy recognized as a critical contributory life-phase. Healthy lifestyle interventions during the antenatal period improve maternal and infant health outcomes, yet translation and implementation of such interventions into real-world health care settings remains limited.

**Objective:**

We aim to generate key implementation learnings to inform the feasibility of future scale up and determine the effectiveness of intervention delivery methods on engagement, experience, acceptability, knowledge, risk perception, health literacy, and modifiable weight-related health behaviors in women during preconception, pregnancy, and postpartum periods.

**Methods:**

This randomized hybrid implementation effectiveness study will evaluate the penetration, reach, feasibility, acceptability, adoption, and fidelity of a healthy lifestyle intervention (OptimalMe) implemented into, and in partnership with, private health care. Individual health outcomes associated with implementation delivery mode, including knowledge, risk perception, health literacy, self-management, and health behaviors, are secondary outcomes. A total of 300 women aged 18 to 44 years, who are not pregnant but wish to conceive within the next 12 months, and with access to the internet will be recruited. All participants will receive the same digital lifestyle intervention, OptimalMe, which is supported by health coaching and text messages during preconception, pregnancy, and postpartum periods. We will use a parallel 2-arm design to compare telephone with videoconference remote delivery methods for health coaching. Methods are theoretically underpinned by the Consolidated Framework for Implementation Research and outcomes based on the Reach, Engagement, Adaptation, Implementation and Maintenance framework.

**Results:**

The study was approved on August 16, 2019 and has been registered. Recruitment commenced in July 2020, and data collection is ongoing. Results are expected to be published in 2022.

**Conclusions:**

The study’s design aligns with best practice implementation research. Results will inform translation of evidence from randomized controlled trials on healthy lifestyle interventions into practice targeting women across preconception, pregnancy, and postpartum periods. Learnings will target consumers, program facilitators, health professionals, services, and policy makers to inform future scale up to ultimately benefit the health of women across these life-phases.

**Trial Registration:**

Australian and New Zealand Clinical Trial Registry ACTRN12620001053910; https://www.anzctr.org.au/Trial/Registration/TrialReview.aspx?id=378243&isReview=true

**International Registered Report Identifier (IRRID):**

DERR1-10.2196/33625

## Introduction

### Background

Obesity is a major global public health and economic burden. In an increasingly obesogenic environment, young women are a high-risk population group with suboptimal lifestyle behaviors [1], accelerated weight gain, and rising obesity prevalence across early to middle adulthood [2,3]. Prior to pregnancy, excess weight affects fertility [4] and independently increases the risk of adverse maternal and neonatal outcomes [5]. During pregnancy, up to 50% of women exceed international gestational weight gain recommendations [6], which increases their risk of complications, such as gestational diabetes, cesarean section delivery, and having a large-for-gestational-age infant, compared with those whose weight gain is within recommendations [6,7]. Independent of maternal prepregnancy BMI, excessive gestational weight gain increases subsequent childhood [8,9] and maternal obesity by 3-fold [10]. Excessive gestational weight gain superimposed on preexisting overweight or obesity further exacerbates risks and perpetuates a cycle of weight gain in women across their reproductive lifespan [11].

Pregnancy is a critical window in which maternal health behaviors and lifestyle should be optimized to benefit the future health of both mother and child [11]. Consequently, most research to date has concentrated on efficacy-based antenatal lifestyle interventions for improving outcomes [12,13]. A recent systematic review and meta-analysis [12] by the US Prevention task force of 68 trials that involved a total of 25,789 women reported a reduction in gestational weight gain after behavioral lifestyle interventions (mean difference −1.02 kg, 95% CI −1.30 to −0.75; 55 studies; n=20,090), with an associated reduction in gestational diabetes (relative risk 0.87, 95% CI 0.79 to 0.95; 43 trials; n=19,752) and emergency cesarean delivery (relative risk 0.85, 95% CI 0.74 to 0.96; 14 trials; n=7520) risks [12]. This level 1 evidence on antenatal healthy lifestyle intervention efficacy is supported by findings of cost-effectiveness and potential cost savings [14], mandating translation into policy and practice [12,15].

However, vital implementation gaps remain [16]. Weight management guidelines for preconception and pregnancy periods lack quality, consistency, and translation of effective intervention strategies into practice with extended reach that is in line with real world experience [17]. Barriers in the preconception period include identifying and engaging women who intend to become pregnant and who are, otherwise, not regularly engaged with the health care system [18]. In pregnancy, identifying broad reach, feasible intervention delivery methods, including remote delivery options, remains unclear [12,13]. Barriers in the postpartum period include engagement, penetration, and uptake of healthy lifestyle interventions with very limited reach and impact to date [19]. To leverage the substantial investment in efficacy trials and deliver health impacts, these barriers must be addressed.

We previously designed a low-intensity, low-cost healthy lifestyle program, called HeLP-her, that has engaged thousands of reproductive-aged women and has an extensive evidence base [16,20-24]. The program effectively prevents progressive weight gain in reproductive aged women [22,23], estimated to be between 0.625 kg and 1.2 kg per year [25], depending on the population studied [2,26]. During pregnancy, HeLP-her optimized gestational weight gain (intervention: mean 6.0, SD 2.8 kg; control: mean 6.9, SD 3.3 kg; *P*<.05) and postpartum weight retention (intervention: mean 0.51, SD 4.48 kg; control: mean 1.96, SD 5.74 kg; *P*<.05) overall, with the greatest efficacy demonstrated in nonobese women [20,21,27]. HeLP-her is theoretically underpinned and improves self-management behaviors through health coaching supported by intervention resources and self-management tools. It has been contextually adapted successfully across delivery methods, settings, and life stages, retaining core components to ensure fidelity [16,20,21,23].

Our formative work has included extensive evidence synthesis to systematically evaluate the efficacy of lifestyle interventions incorporating diet, physical activity and weight- and self-management behaviors during preconception [28], pregnancy [13], and postpartum [19] periods, to integrate key intervention components and inform study design. We have developed and integrated health-related content based on best practice clinical guidelines [29,30] and have identified facilitators and barriers to healthy lifestyle– and weight-related behaviors, information preferences, and health professional engagement across these life stages [31-34]. We have engaged consumers and health professionals to iteratively adapt our evidence-based intervention for broader reach, with translation of the intervention content, resources, and tools to a dedicated web-based digital platform, and have performed extensive consumer testing to evaluate and iteratively optimize acceptability, relevance, and engagement of intervention content in a representative target population of women.

### Overall Aim

Applying a hybrid effectiveness-implementation study, we aim to generate key implementation learnings to inform the feasibility of future scale up and determine the effectiveness of intervention delivery methods on engagement, experience, acceptability, knowledge, risk perception, health literacy, and modifiable weight-related health behaviors in women during preconception, pregnancy, and postpartum periods.

### Specific Objectives

Our objectives are as follows:

1. Determine implementation feasibility with 1) process evaluation (ie, measure of process used to implement the program and any variation experienced; facilitators and barriers to intervening events impacting implementation), 2) the RE-AIM framework to assess Reach, Effectiveness, Adoption, Implementation and Maintenance of the intervention and 3) cost effectiveness analysis.
2. Evaluate intervention participation (ie, engagement and adherence to program) and engagement (ie, degree of online module completion, frequency, and duration of time spent on the platform).
3. Determine intervention effectiveness on health related outcomes measured at the individual level including health knowledge, health literacy, and self-management behaviors.
4. Determine any discrepancy according to the health coaching delivery method.

### Hypotheses

We hypothesize as follows:

1. The intervention will be feasible to implement and can effectively reach and engage women prior to pregnancy through co-designed strategies that are acceptable to women and the implementation partner with demonstrated cost-effectiveness.
2. Participation and engagement with intervention resources will be greater for participants who complete the intended intervention dose compared with those who do not.
3. The intervention will improve preconception and pregnancy health knowledge and self-management.
4. Phone and videoconference health coaching delivery will be equally feasible and cost-effective, yet engagement, adherence, and effectiveness will be greater with videoconference compared with phone-based health coaching.

## Methods

### Implementation

#### Design

OptimalMe is a type 3 hybrid effectiveness-implementation study [35] with an active intervention phase (2 years) and a passive observation phase (up to 5 years). Type 3 hybrid implementation designs are those in which implementation outcomes are primary, and individual or population outcomes are secondary [35]. The primary outcome of the project includes overall intervention penetration and reach and the feasibility, acceptability, adoption, and fidelity of the delivery of OptimalMe. Secondary outcomes include evaluation of individual health outcomes associated with implementation delivery mode, including knowledge, risk perception, health literacy, self-management, and health behaviors. The study design is a parallel, two-arm, randomized trial at the level of the individual utilizing a pragmatic philosophy, working within real-world conditions to assess overall effectiveness. All individuals will receive the same evidence-based lifestyle intervention, and implementation delivery methods will be compared.

#### Setting

The Australian health care system is government supported via Medicare, which provides universal free care to Australian citizens and residents (and others who are eligible) and is supported by a subsidized private health system. Private health insurance, paid by the individual, allows patients to choose hospitals and health care providers from outside of the public system, with a 12-month waiting period before some, or all, of the cost of hospital treatment is reimbursed. Overall, approximately 54% of Australian adults have a form of private health insurance [36], and 27% of births in Australia occur in private hospitals [37]. Women who give birth in private hospitals attend ambulatory private obstetric care and have limited contact with hospitals prior to delivery.

Women who upgrade private insurance to include, or join with, pregnancy and birth coverage, comprise a unique population, prospectively signaling future intention for a pregnancy. In a sample of 294 women who had recently upgraded or obtained insurance for pregnancy and birth coverage, 41% intended to conceive within the next 12 months [33].

The implementation partner in this research program is Medibank Private, which is one of Australia’s largest insurers (funding approximately 20,000 births annually). Feasibility scoping shows approximately 7800 women join with, or upgrade to, pregnancy and birth coverage with this insurer nationally each year.

#### Framework

This implementation research is underpinned by the Consolidated Framework for Implementation Research [38], which provides a pragmatic framework, informed by *translation into practice* theories, that is designed to guide complex implementation projects and generate knowledge across settings and studies [38]. The framework consists of 5 domains [38]: Domain 1 consists of the unadapted intervention to be implemented and assumes the intervention is composed of core or fundamental components, essential to efficacy, surrounded by peripheral components that are adaptable to the local context, without altering the integrity of the intervention. The adaptable components are informed by domain 2 (the outer setting, ie, policy, guidelines, population needs), domain 3 (the inner setting, ie, the organization’s structure, culture, readiness to change), and domain 4 (the individuals within the outer or inner setting involved in the intervention as influencers of implementation. The implementation process (domain 5) works across all domains to achieve implementation through an iterative change process of executing and evaluating implementation activities [38].

The fundamental core components of our intervention include theoretical underpinning; simple diet; physical activity and self-management messages; low-intensity delivery format; individual health coaching sessions focused on goal setting, problem solving, and self-management delivered by a qualified health professional; and ongoing intervention support via text messaging (Domain 1). Core components were informed by our extensive intervention evidence base [16,20,21,23] and were consistently applied to setting, population, intervention tools, resources, and delivery method and format. The integration of peripheral intervention components was undertaken to incorporate best practice clinical guidelines and systematic review lifestyle intervention evidence (Domain 2) and using an intervention co-design process with the implementation partner, Medibank Private (Domain 3) experts in obstetric and lifestyle delivery and reproductive women in this life stage (Domain 4). This included the incorporation of health education resources within the intervention and the development of a consumer-tested web-based digital platform for remote delivery and comanaged participant engagement. The efficacy of health coaching delivery methods (phone and videoconference) will be compared. A governance process has been established to enable responsive and pragmatic adaptations to peripheral components in partnership with the implementation partner (Medibank Private), yet designed and managed by the clinician academic research group (Monash University). The primary outcomes form Domain 5, which includes overall intervention feasibility, reach, acceptability, and adoption as well as fidelity of the delivery of OptimalMe as planned.

### Study

#### Design

The study will be conducted in accordance with Consolidated Standards of Reporting Trials [39] and Template for Intervention Description and Replication frameworks [40].

#### Eligibility Criteria

The target intervention population will include Medibank Private members who have joined or upgraded with pregnancy and birth coverage within the 3 months prior to recruitment (to align with likely planned conception based on insurance uptake and wait times). Eligibility criteria focus on inclusiveness and includes those who are not pregnant, who wish to conceive within 12 months of recruitment, who are aged 18 to 44 years, with any BMI, who read and speak English, and who have access to an internet-capable device will be included.

#### Sample Size

Given the implementation effectiveness study design, sample size has not been powered on a clinical outcome because the primary outcome is to determine implementation learnings to inform feasibility. Available funding enables intervention delivery to approximately 300 women, which is approximately 10% of the eligible population with intention to conceive, based on our formative research [33].

#### Randomization

Participants will be randomized to receive health coaching either by telephone or via videoconference. An external senior statistician will provide computer-generated randomization codes to the research coordinator only, who will sequentially allocate all participants but will have no role in intervention delivery. Researchers involved in intervention delivery and data collection will be blinded to the allocation sequence; however, due to the nature of the intervention, they will not be blinded to participant allocation. Researchers responsible for data analysis and reporting will be blinded to both allocation sequence and participant allocation. Randomization performed external to the implementation setting is designed to reduce bias. Due to the nature of the intervention, participants will not be blinded to group allocation.

#### Recruiting Strategy

A co-designed process, using an opt-in design, was developed with the implementation partner to facilitate Australia-wide recruitment. We will use direct email communication (approximately 500 members every month, to be varied based on response rates and historical trends in email engagement observed during specified periods, including seasonal holiday periods) to recruit women (randomly selected to receive a targeted invitation by system generated mailing lists) who meet initial eligibility criteria (insurance coverage and age) with a link to the landing page of the web-based intervention platform. The page contains introductory information about the healthy lifestyle intervention, including a video. Individuals who wish to take part will be required to confirm remaining eligibility criteria, including pregnancy status and intention to conceive within 12 months, provide informed consent electronically, and register via a digital interface. The researcher coordinator will then contact the Medibank-managed integrated voice recognition system to confirm the potential participant’s membership (using first and last name, membership ID, date of birth, and postal code) and pregnancy and birth coverage status to confirm eligibility. An email with an activation link to an account for the intervention will be sent to participants, at which time, they are randomized to 1 of the 2 coaching delivery methods. Recruitment will continue until target numbers (n=300) are reached. This pragmatic approach enables management of participant flow into the intervention and will not disqualify those who may be unaware of pregnancy status at point of recruitment or who may re-evaluate their intention to become pregnant after recruitment (Multimedia Appendix 1).

### Intervention

#### Theoretical Underpinning

This intervention is based on our previous healthy lifestyle program—HeLP-her [16,20-23,27]). HeLP-her is a low intensity, behavior change program, grounded in social cognitive theory [41]. HeLP-her is nonprescriptive and provides health coach–delivered simple messages on healthy lifestyle behaviors aligned with national dietary and physical activity guidelines [30,42,43]. These are reinforced by behavior change strategies including identifying individual health priorities and facilitators and barriers for change. Realistic achievable goals are prioritized and developed by participants, and a behavioral action plan that outlines goals and timeframes is established. Individual barriers, strategies for change, and social supports are identified and discussed, and self-monitoring is practiced and encouraged [27].

#### Delivery

**Figure 1:**
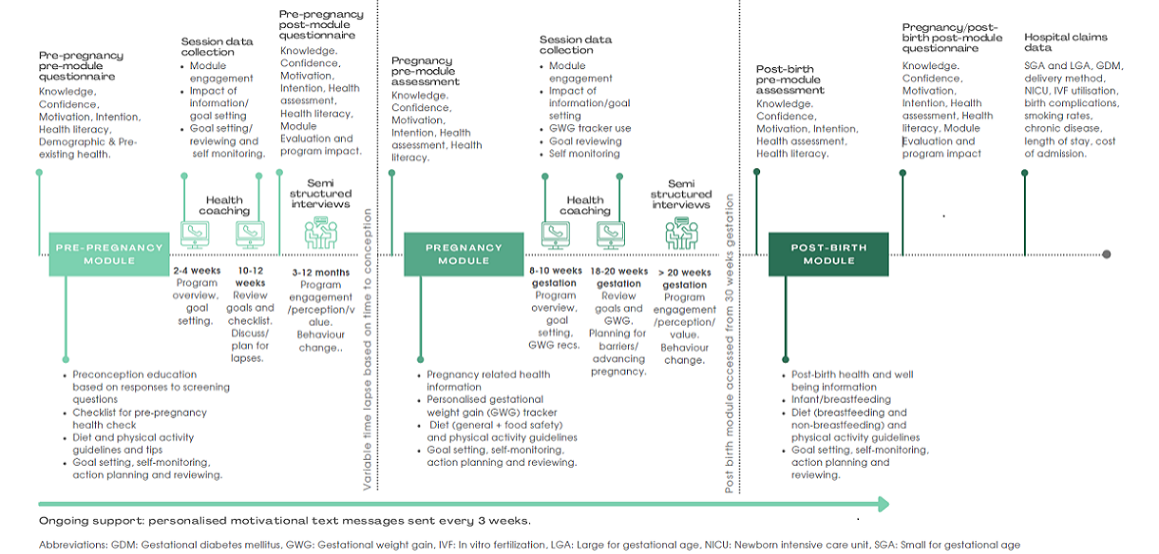
OptimalMe program design and delivery.

Co-design of intervention delivery with the implementation partner prioritized remote delivery to ensure equitable accessibility to the intervention across Australia by using a dedicated web-based digital platform, supported by health coaching (delivered via phone or videoconference), with ongoing text message support (Figure 1).

#### Platform

**Figure 2:**
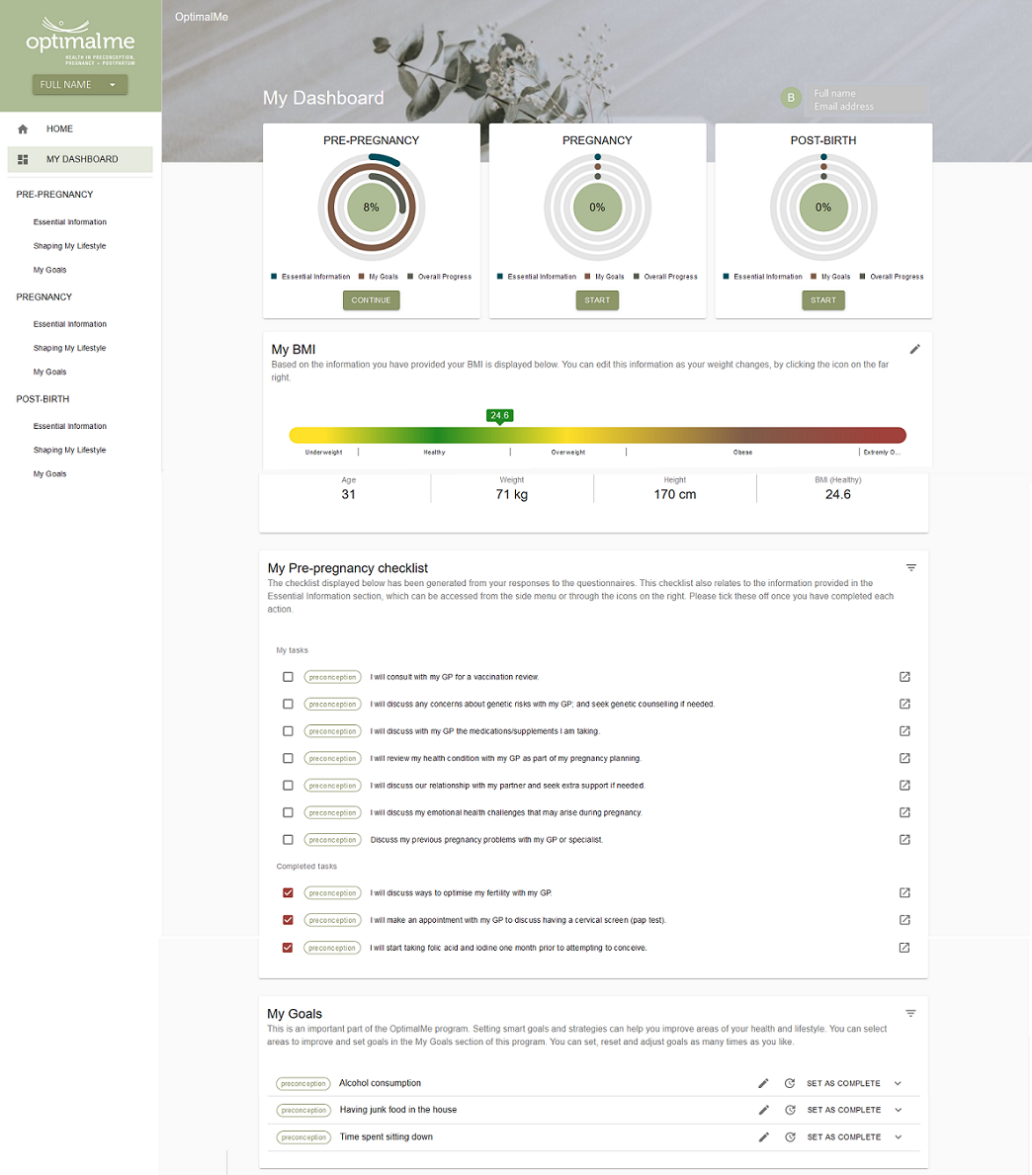
Platform user dashboard.

The secure web-based platform can be accessed via desktop and mobile apps. The platform contains preconception, pregnancy, and postpartum modules. Participants are provided access to the preconception module at the outset from the dashboard (Figure 2). The pregnancy module is accessible to participants when they update their personal profile on the dashboard (pregnancy status and estimated due date). In addition to the 3 modules, the dashboard contains an interactive BMI (and gestational weight gain, if pregnancy is reported) calculator, a checklist, and activities to review behavioral action plans.

All modules have a similar format—each has a health information (education) section and a healthy lifestyle behavior change (self-management) section. The health information section contains health, medical, and screening information and the healthy lifestyle section contains diet and physical activity recommendations, as well as an interactive behavior change section. Health, medical, and screening information are presented as a suite of fact sheets specific to each reproductive phase (Multimedia Appendix 2). Information provided is based on the Royal Australian College of General Practitioners Red Book [29], Australian Government Clinical Practice Guidelines for Pregnancy [30], and our formative research [33,34]. A series of health screening questions at module commencement based on these guidelines will inform the presentation of fact sheets according to relevance. For example, preconception participants will be asked when their last cervical screen was completed. If a participant indicates a cervical screen outside of a guideline-specified timeframe, the relevant cervical screening fact sheet will be presented under *Essential information* at the top of the screen. Conversely, if a participant indicates a cervical screen within a guideline-specified timeframe, the fact sheet will be presented under *Other recommended reading*. This design feature ensures that participants are directed to the information that is most relevant to their health needs (based on their responses to screening questions) while minimizing the burden of information, which has previously been identified as a barrier to receiving health information [31].

Fact sheets have a similar structure—each fact sheet has 3 to 4 key messages, followed by detailed topic information and links to other websites for additional evidence-based information. Each fact sheet is supported by an interactive component that enables the user to populate a checklist item summarized on the platform dashboard (Figure 2). For example, opening a fact sheet about cervical screening will populate the *Check my cervical screening status with my GP* item. Health literacy is tested at the top of each fact sheet with a true or false question, with corresponding information that explains the correct answer.

Healthy lifestyle resources include fact sheets related to weight gain prevention and infographics that are based on Australian adult pregnancy [30] and breastfeeding [42] dietary guidelines and physical activity guidelines [43]. Additional resources include information on how to read food labels, how to estimate food portion sizes, healthy snack and food substitution ideas, and calories consumed using various food choices versus those burned from walking. Behavior change is supported by an interactive goal-setting section that guides the user to develop a personalized goal through action planning, which includes an activity to self-select modifiable health behaviors for improvement (eg, packaged or convenience food consumption, alcohol intake, physical activity, fruit and vegetable intake, and sleep). Areas of relevance are selected and prioritized by each participant (ie, areas are ranked in order of importance). Participants are guided through goal-setting using free text to specify what they would like to achieve identify motivating factors and social support pathways, barriers to behavior change and specific strategies to overcome barriers that are time dependent and identification. An action item to review goals will be automatically added to the digital platform, which is encouraged 2 weeks after goal planning and commitment (Figure 2). 

The platform was consumer-tested using a quantitative survey for functionality (ie, ease of navigation across the platform), acceptability (ie, usefulness of the information, presentation and aesthetics, ease of understanding content) and relevance (ie, appropriateness of information, potential for the platform to assist in optimizing health behaviors, peer recommendation). The survey contained a series of statements requiring response on a 5-point Likert scale that ranged from strongly disagree to strongly agree, with an opportunity to provide free text. Responses were transformed to a binary representation (0, disagree and neutral; 1, agree). Overall, 36 women were recruited from the community with advertisements across all modules using both computer (19/36, 53%) and mobile phone or tablet (17/36, 47%) devices. Women were aged between 25 and 38 years old, and the majority were university educated (33/36, 92%). Most women agreed that they could navigate to different areas of the platform and return to the dashboard with ease (23/30, 77%); they found the platform to be aesthetically appealing (25/36, 69%), and the amount of information to be appropriate (28/36, 78%) and easy to understand in its presentation (29/30, 97%). The majority believed that the information would assist them in improving their health-related behaviors (25/30, 83%) and considered the platform relevant to recommend to women of the same life stage (24/36, 67%).

#### Health Coaching Sessions

Health coaches with a tertiary qualification in health sciences (ie, dietetics, nutrition, exercise physiology, or allied health) will deliver the program and aim for continuity wherever possible to maintain rapport. The purpose of the individual health coaching sessions is to build rapport with participants, reiterate program objectives, enhance engagement, practice goal setting and self-management skills, support participants with lifestyle modules, and provide personalized feedback on behavior change. Any module components that have not been accessed or completed by participants at the point of health coaching will be flagged for completion by the participant during or after the session.

All participants will be offered 2 personalized preconception health coaching sessions (approximately 20 minutes in duration, delivered either be phone or videoconference according to randomization) 2 to 4 weeks after program entry and 6 weeks later.

During pregnancy, 2 additional 20-minute health coaching sessions will be scheduled (8-10 weeks gestation or 1-2 weeks after starting the pregnancy module commencement and 19-20 weeks gestation).

#### Ongoing Program Support

SMS text messages will be sent every 3 weeks as a reminder to practice healthy behaviors.

### Fidelity

Intervention fidelity will be maintained by facilitators using a checklist after health coaching sessions to reduce potential reporting bias. The checklist will include planned discussion points, deviation in delivery of session with reasons, and duration of session. Coaching sessions will be periodically recorded with participant consent to monitor fidelity.

Intervention facilitators will complete program-specific training on the intervention, including health coaching delivery. Facilitators will be required to have a sound knowledge of evidence-based practice; an understanding of health behaviors, nutrition, and physical activity; and a tertiary qualification in a health-related discipline. Program-specific training includes both theory and practical components and motivational interviewing techniques [20-23,44].

### Outcome Measures

Outcome measures (Table 1) are underpinned by the RE-AIM (Reach, Effectiveness, Adoption, Implementation and Maintenance [45]). Both quantitative (recruitment and intervention delivery fidelity checklists [46]) and qualitative (semistructured interviews) data collection methods will be used (Multimedia Appendix 1 and Multimedia Appendix 2).

**Table 1:** Description of outcome measures.

Outcome			Description
**Implementation feasibility (primary)**			Program evaluation and feasibility for future scale up
	Reach	Proportion of the target population that were invited and participated in the program and intervening factors	
	Implementation fidelity	Delivery according to design and any variation experienced Facilitators and barriers: identification and description of intervening events	
	Adoption of the program by the implementation partner	Contextual events or factors influencing implementation within the setting, variation in any co-design implementation component	
	Cost-effectiveness	To answer questions about overall feasibility of implementation	
**Intervention effectiveness (secondary)**			Exploratory evaluation of the effectiveness of intervention delivery across preconception and pregnancy (Figure 1)
	Participation	Adherence and engagement measures to intervention dose including health coaching sessions and web-based platform interaction including degree of module completion, frequency and duration of time spent on the platform	
	Acceptability	A set of questions relating to the influence of the program in changing health behaviors, the usefulness and relevancy of the information provided, valuable aspects of the program and areas for improvement Qualitative data analysis of insights, participation factors, intervention reach, adoption and maintenance of behavior change, intervention delivery format, and areas for improvement until thematic data saturation	
	Effectiveness	On individual health behaviors including self-reported weight, health literacy [47], self-management [48], diet [49], and physical activity [50] using validated questionnaires Collected at the start of the intervention, after preconception health coaching sessions and module, and at the start of the pregnancy module	
	Pregnancy and birthing outcomes	In vitro fertilization utilization (restricted to only hospital component visibility of this process such as retrievals and transfers); gestational diabetes diagnosis, delivery type (ie, vaginal or cesarean section), birth complications and neonatal intensive care unit admission, length and cost of hospital stay and ancillary utilization (ie, physiotherapy, dieticians, dental) Captured via encrypted data linkage with Medibank Private for health outcomes up to and including 5 years after the start of the study as observational study phase data	

### Statistical Analysis

Deidentified quantitative and qualitative databases will be maintained on encrypted Monash University servers and managed by research staff involved in data collection. We will use descriptive approaches to evaluate primary outcomes measures. Quantitative data collected for secondary outcome measures will be exported to STATA (version 17.0; StataCorp LLC). Descriptive statistics (means with standard deviations or frequencies with ranges) will be used to characterize intervention effectiveness and the recruited sample by demographic characteristics (age, BMI, country of birth, education, socioeconomic status, and parity), preexisting health, and health-related behaviors (ie, self-management, diet, and physical activity). Logistic and linear regression models will be used to explore associations between before and after the intervention. Additionally, factors known to influence secondary outcomes, including weight, such as diet, physical activity, breastfeeding status, and parity will be adjusted for a priori. Mixed-effects regression models, with the individual specified as the random effect, will be investigated to account for repeated measures. Missing data will be examined, and multiple imputation will be used to generate complete data, if data are not found to be missing at random. Sensitivity analyses will be performed to explore robustness. A *P* value <.05 will be considered statistically significant.

Transcripts of semistructured interviews will be independently analyzed and coded by 2 researchers using NVivo software (version 12; QSR International). Data will be searched for concepts in relation to participatory factors and program evaluation, with codes generated and grouped into themes using an inductive approach. Quantitative data will be analyzed first, to inform thematic analyses. The definitions of themes will be determined by consensus (between 2 researchers).

#### Economic Evaluation

The economic evaluation will be designed to identify the costs associated with implementation, and the net costs to health care funders. Costs of the OptimalMe implementation package will be identified from the trial data, including the costs of platform maintenance, staff time (in providing the coaching sessions), and SMS text messages. Fixed and variable costs will be identified, allowing cost per woman to be estimated at different scales of the intervention. The net costs to health care funders will be identified by quantifying the costs associated with birth type, birth complications, neonatal intensive care unit admissions, hospital stay, and ancillary utilization. Costs to Medicare Benefits Schedule will be identified based upon item numbers [51] associated with birth type and complications. Costs to private health insurers associated with hospital stay will be identified directly from the study. Costs to public hospital funders from neonatal intensive care unit admissions or any public hospital transfers will be identified from the National Hospital Cost Data Collection produced by the Independent Hospital Pricing Authority [52]. The total cost per woman will be calculated, and generalized linear models will be utilized to identify differences in costs between delivery methods. We will use these models to estimate the net cost impacts to different funders at different levels of the population reached.

### Ethical Approval

The Monash Health Human Research and Ethics Committee approved the study (RES-19-0000291A), and the study has been registered (using predefined study description classifications; as such, the trial was registered as an efficacy trial in the absence of a feasibility study descriptor) on the Australian and New Zealand Clinical Trial Registry (ACTRN12620001053910).

## Results

The project is supported with funding from Medibank Private Ltd. Recruitment commenced in July 2020 with results expected to be published in 2022.

## Discussion

Prevention of weight gain and obesity is a global health priority. Increased emphasis is placed on high-risk populations [53,54], including reproductive-age women with accelerated preconception, pregnancy, and postpartum weight gain [6]. Lifestyle interventions can be used to optimize weight and reduce maternal and neonatal adverse outcomes [12,55], yet translation of effective interventions into real-world settings remains critically limited. We address this gap and leverage extensive investments in efficacy research by undertaking implementation research to inform feasibility, acceptability, applicability, effectiveness, and sustainability of an evidence-based weight gain prevention intervention for preconception, pregnancy, and postpartum periods. Implementation research leverages investment in efficacy-based randomized trial knowledge to study methods that promote the systematic uptake of evidence-based interventions into practice and policy to improve health [35].

Our study design aligns with best practice implementation research; focuses on system-level outcomes; and is underpinned by evidence from efficacy trials, systematic reviews, meta-analyses, and guidelines. Additional health information, specific to preconception, pregnancy, and postpartum life stages, has been integrated, with checklists and resources. Evidence on core and peripheral components has been integrated to adapt the intervention with stakeholders across women, multidisciplinary clinicians, and partners. Novel delivery strategies, including sophisticated digital platform and remote health coaching delivery methods, while retaining core intervention features including low-intensity individual health coaching and ongoing text message support. This work has integrated, and been supported by, robust implementation and intervention frameworks and theories.

We anticipate that the OptimalMe intervention will demonstrate feasibility and directly provide evidence to inform scaled intervention delivery. Learning will not only inform future implementation design but translation of evidence targeting consumers, program facilitators, health professionals, services, and policy makers to inform future scale up of healthy lifestyle interventions to ultimately benefit the health of women.

## Appendix

Multimedia Appendix 1 Co-designed recruitment strategy.

Multimedia Appendix 2 OptimalMe health-related content (fact sheets) within preconception, pregnancy, and postpartum modules.

Multimedia Appendix 3 CONSORT eHEALTH Checklist (V 1.6.1).

## Abbreviations

BMI: body mass index
