# Supplementary figures and images for: OptimalMe Intervention for Healthy Preconception, Pregnancy, and Postpartum Lifestyles: Protocol for a Randomized Controlled Implementation Effectiveness Feasibility Trial
Source: JMIR Res Protoc. 2022 Jun 9;11(6):e33625. doi: 10.2196/33625 (PMC9227652; doi:10.2196/33625)

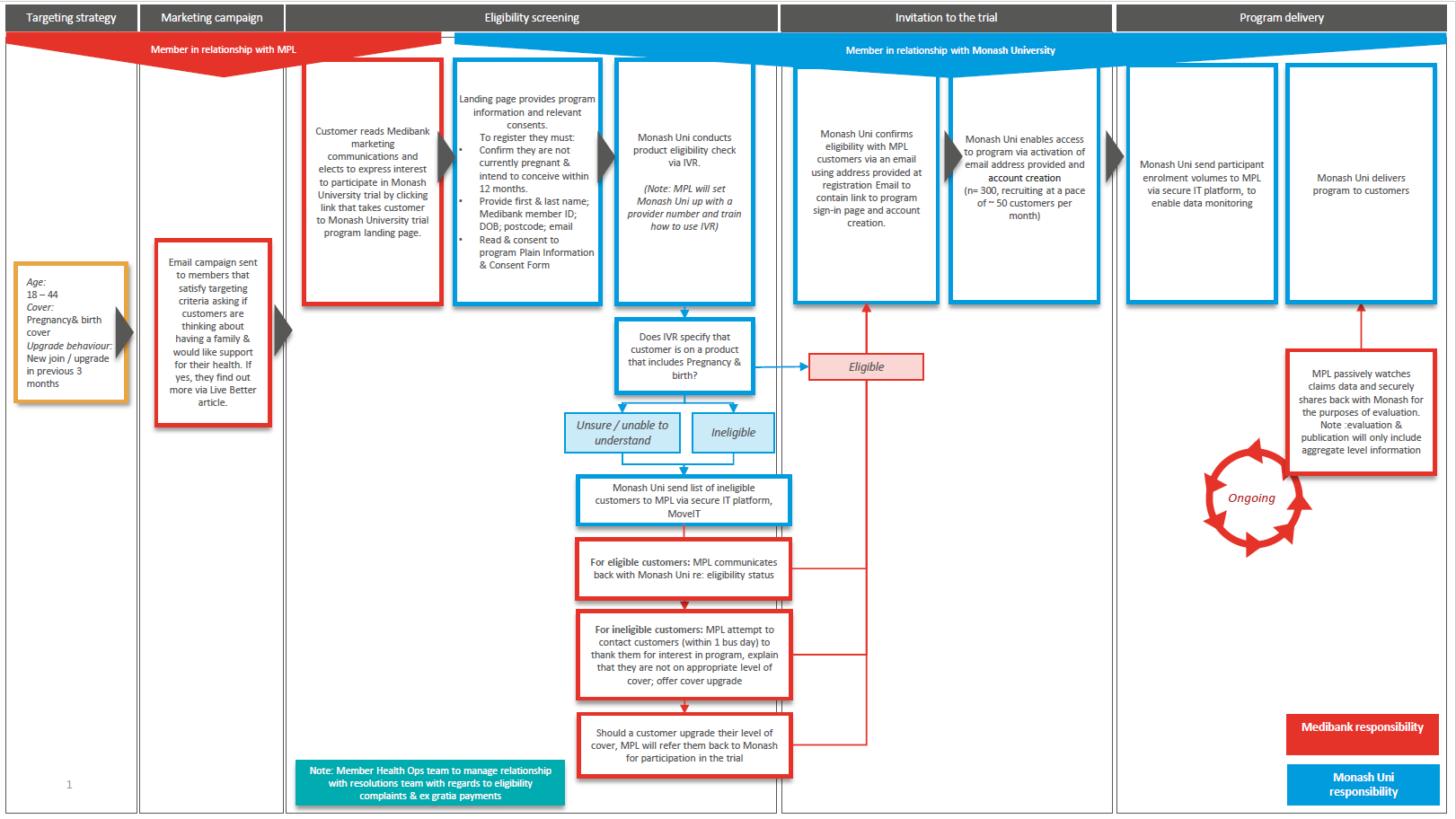

Supplement: Multimedia Appendix 1 [file resprot_v11i6e33625_app1.png]

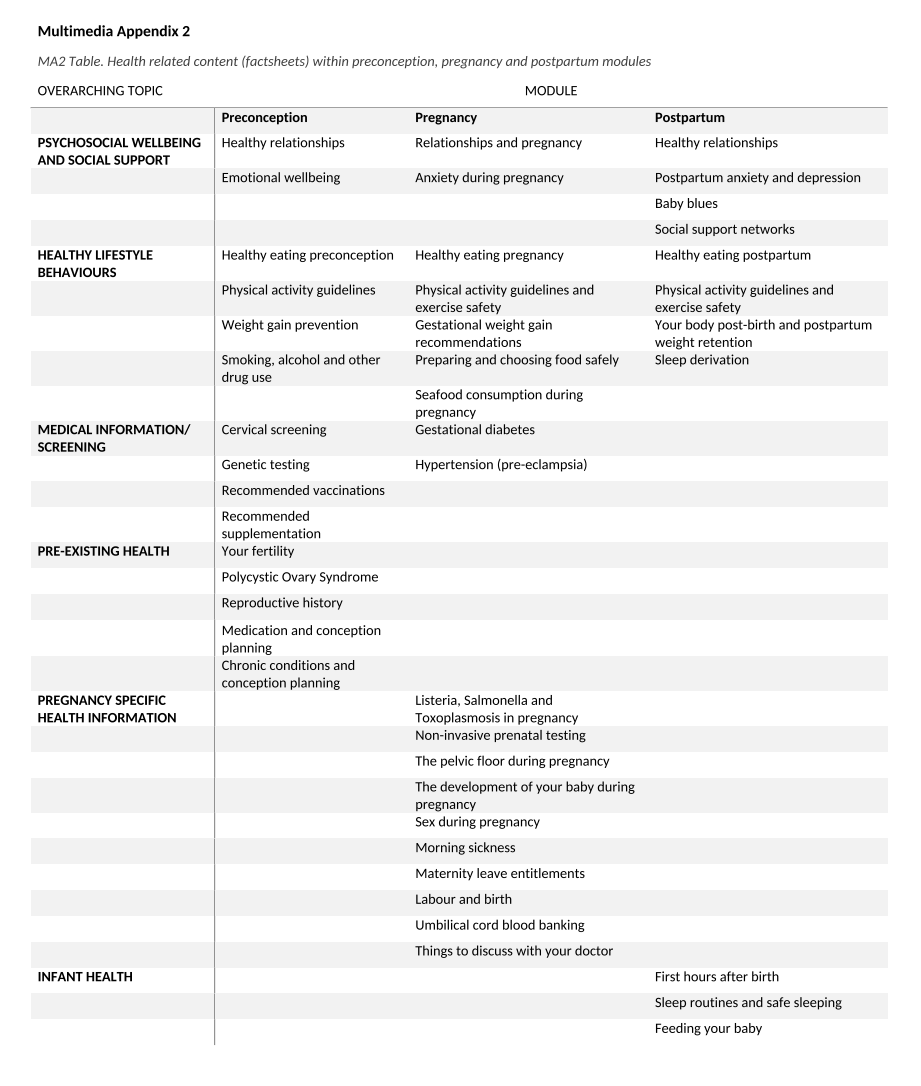

Supplement: Multimedia Appendix 2 [file resprot_v11i6e33625_app2.png]
